# Geoenvironmental characteristics of bisphenol A contaminated soil after persulfate treatment with different activation/enhancement methods

**DOI:** 10.1371/journal.pone.0214024

**Published:** 2019-04-18

**Authors:** Fuming Liu, Yong-Zhan Chen, Shuping Yi, Wan-huan Zhou, Linshen Xie, Hongyun Ma

**Affiliations:** 1 School of Environmental Science and Engineering, Southern University of Science and Technology, Shenzhen, China; 2 Department of Civil and Environmental Engineering, Faculty of Science and Technology, University of Macau, Macau, China; 3 State Key Laboratory of Internet of Things for Smart City, University of Macau, Macau, China; 4 Guangdong Provincial Key Laboratory of Soil and Groundwater Pollution Control, Shenzhen, China; 5 Shenzhen Institute of Environmental Science, Shenzhen, China; 6 Key Laboratory for Groundwater and Ecology in Arid and Semi-arid Areas, CGS, Xi’an, China; CAS, CHINA

## Abstract

Persulfate (PSF) is a strong oxidant that has been used extensively in the In-Situ Chemical Oxidation (ISCO) technology. The geoenvironmental impact of PSF treatment is barely investigated. This situation should be carefully considered as it may affect the reutilization of contaminated soil as engineering materials. This paper studied the removal of bisphenol A (BPA) by PSF with Nano Zero-Valent Iron (nZVI) and percarbonate (SPC) activated/enhanced and their subsequent impacts on the engineering properties of soil. The physicochemical and geotechnical properties of soils before and after treatment were evaluated using batch experiments. The results indicate that the introduced pristine PSF can be activated by some naturally occurring matters and subsequently lead to the mineralization of BPA. Both non-activated PSF and activated/enhanced PSF treatment led to the soil improvement in the undrained shear strength at different degrees. The primary mechanism of soil improvement is ascribed to the heterogeneous sulfate and/or carbonate precipitation. Meanwhile, Ca^2+^ in the pore fluid played a significant role in the enhancement of the soil strength. A conclusion was drawn that the treatment of both non-activated PSF, nZVI- and SPC-activated PSF treatment can achieve removal of BPA and soil improvement in the short-term simultaneously. This study can improve the PSF-involved remediation of brownfields and dredged sediments for a sustainable and low-carbon society.

## 1 Introduction

Soil is a fundamental resource in the works of foods, raw materials, and bio-based energy. However, increasing soils are contaminated by various contaminants from varieties of anthropogenic activities. Extensive efforts have been devoted to developing soil remediation technologies. Among them, In-Situ Chemical Oxidation (ISCO) is efficaciously a cost-effective remediation method that has been implemented extensively to cleanup organic contaminants in soils and groundwater. ISCO aims directly to mineralize organic contaminants into harmless or biodegradable products such as carbon dioxide (CO_2_), carbonate (CO_3_^2-^), and water. Among different oxidants used in ISCO, the technologies based on persulfate (PSF) are rising approaches for the removal of recalcitrant organic contaminants in soils and groundwater [1, 2].

The mechanism of PSF treatment in the removal of organic contaminants is like the way in which hydrogen peroxide (H_2_O_2_) converted to hydroxyl peroxide: with appropriate stimulation, the sulfate radical will be generated [3]:

$$S_{2}O_{8}^{2-}+stimulation={SO}_{4}^{-}\cdot +({SO}_{4}^{-}\cdot \;or{\;SO}_{4}^{2-})$$ (1)

Sulfate radical (SO_4_^−^·) is a strong one-electron oxidant with a high redox potential of 2.5–3.1 V and is nonselective for mineralization of a range of organic compounds [4]. The stimulation, also called activator, has been investigated in aspect of UV [5], heat [6], base [7], transition metals [8], minerals [9], sonochemistry [10], citric acid [11], electrochemical [12], nanocarbons [13], and quinones [14]. The generated sulfate radical can spontaneously and rapidly oxidize organics and ultimately transform to sulphate ion as the by-products [15]:

$${SO}_{4}^{-}\cdot +organic\to {SO}_{4}^{2-}+{CO}_{2}+H_{2}O+\;intermediate(s)$$ (2)

In addition to the mineralization of organics, the increment of the deuterogenic sulphate anion can be a potential threat to the human beings and ecological system [16, 17]. For esthetic reasons, sulphate ion has been listed as a secondary maximum contaminant level of 250 mg/L under the secondary drinking water standards by the US EPA [18].

During the past decades, PSF involved ISCO has widespread field application for the cleanup of organic contaminants both in soils and groundwater by injection of PSF and activator slurry into the subsurface, particularly in the remediation of brownfields. Brownfields constitute a substantial land resource in the urbanization of cities, in which many of them have been planned for many efficient uses including commercial buildings, high-rise residential building, and culture/sport and children’s park [19]. For the remediation of contaminated site using PSF treatment, there is a growing civil engineering concerning on its application as the sulfate attach is one of the most aggressive environmental deuteriations that poses threat to the long-term durability of underground structures. This may subsequently generate unnecessary expense or cause some potential damage to the redevelopment of the contaminated soils. Saussaye et al. (2013) confirmed that sulfate ions can induce a significant swelling and decrease of strengths for three different soils after quicklime, cement, and hydraulic road binder treatment [20]. Similar adverse effect of anions, including sulfate, chloride, nitrate and phosphate on the soil strength are also recorded in several publications [21–24]. Liu (2018) suggested that the disturbing component to the soil strength derived by intruding anions are generally dependent on the soil composition, the treatment, the chemical form of the anion, concentration level of contaminants, the cure conditions and the type of geotechnical test [25].

For contaminated site to be remedied, particularly for those planned to be reused, the soil strength and stability are critical for the subsequent constructions [26, 27]. The microscale variation of soil as a result of contamination and the remediation of soil using PSF treatment could affect the geotechnical properties and may ultimately bring about the engineering failure [28, 29]. Unfortunately, little is known about the effect of PSF treatment on geotechnical properties regarding on the reduction of PSF and oxidation of organic contaminants. On the other hand, the disposal of large amounts of municipal solid wastes and sediments dredged from coast, lake, and river is a new environmental problem around the world [30]. After the cleanup of contaminants, they could become available to engineering purposes, for example to recycle contaminated sediments into eco-friendly paving blocks [31, 32]. Therefore, it is significant to investigate the effect of PSF treatment on the geoenvironmental properties of contaminated soil.

In this study, we present a study of the effect of PSF treatment on the geoenvironmental characteristics of clayey soil. Particularly, one of ubiquitous Endocrine Disruptor Compounds (EDCs) well-known for its disturbance of endocrine and ubiquity in environmental medium [33], i.e., bisphenol A (BPA), was spiked in the clayey soil. Nanoscale Zero-Valent Iron (nZVI) and sodium percarbonate (SPC) were used as he activator/enhancement to PSF treatment for the degradation of BPA. These two combined Advanced Oxidation Processes (AOPs), namely nZVI-PSF and SPC-PSF systems, have been extensively investigated and applied in the cleanup of contaminants in soils and groundwater [34]. The geoenvironmental characteristics including removal efficiency of BPA, particle size distribution, soil buffering capacity, cations in pore fluid, mineral component, and vane shear strength were analyzed.

## 2 Methodology

### 2.1 Soil preparation

The soil used in this study was collected near the airport in Macau, China (N22°9'15.84", E113°34'36.76"). The collection of soil samples was approved and guided by Environmental Protection Agency of Macau (Direcção dos Serviços de Protecção Ambiental de Macau) and the relevant regulatory body concerned with the construction waste landfill site. The water content of the soil was firstly adjusted to approximately 85% using distilled water, after which bisphenol A (BPA, (CH_3_)_2_C(C_6_H_4_OH)_2_) at the concentration level of 100 mg/kg (dry soil mass, similarly hereinafter) were spiked to the soil with a vivid agitation to achieve a homogeneous soil slurry. To reach an equilibrium of absorption-desorption of BPA, the spiked soil slurry was sealed and matured for two weeks prior to further treatment. Subsequently, the soil slurry was undergoing pre-consolidation in a vessel with an effective consolidation pressure of approximately 150 kPa. Details of the container can be found in the report by Indraratna et al. [35]. All the soil slurry was mechanically blended for five minutes prior to the above pre-consolidation. The soil samples were obtained when its vertical settlement is stable.

For the preparation of PSF treated soil, different strategies were regarded: 1) 1% PSF (at the mass ratio of dry soil, similarly hereinafter); 2) 7% PSF; 3) 1% PSF + 0.5% Nanoscale Zero-Valent Iron (nZVI); and 4) 1% PSF + 0.5% sodium percarbonate were added to the soil slurry, respectively, prior to the pre-consolidation of the BPA-contaminated soil. Accordingly, these soil samples were symbolized as 1% PSF, 7% PSF, nZVI activated, and SPC enhanced, respectively. The average diameter of the used nZVI is approximately 50 nm, with a specific surface area of approximately 30 m^2^/g (commercially provided and informed by the manufacturer).

### 2.2 Geoenvironmental tests

To evaluate geoenvironmental characteristics of soil samples, the BPA concentration, Atterberg limits, vane shear strength, soil particle size, buffering capacity, pore cations chemistry, and X-Ray diffraction were measured. The BPA concentration level was determined according to the protocols EPA 3550 C 2007 using Agilent 1260 Infinity. The soil was freeze dried prior to the measurement. The laser diffraction technique (i.e., Mastersizer-3000 Malvern) was employed to characterize the soil particle size using Mastersizer 3000 (Malvern) in deionized water solution under electric (3000 rpm) and ultrasonic (40W, 40kHz) mixing. The soil buffering capability was evaluated by the following method: 10 g of dry soil were added in 200 mL of deionized water, then the turbid solution was stirred, and ultrasound treated for 10 min. After that, 100 uL 5 mol/L hydrochloride acid was added at an interval time of 5 min, until the pH value reached approximately 2. The typical cations concentration level in soil pore fluid was determined as following procedure: take 1 g soil in 25 mL ultrapure water and vibrated for 24 h. Then, the soil solution was centrifugated at 4000 rpm for 20 mins. The obtained supernatant liquor was passed 0.22 um filter membrane and determined on the Inductively Coupled Plasma Mass Spectrometry (ICP-MS, Agilent 7700x). Particularly, nitrate acid (65% in volume fraction, similarly hereinafter) was added to each supernatant liquor to about 5% prior to the determination.

The mineral components of soil samples were analyzed using X-Ray powder diffraction (XRD) on the Rigaku Smartlab diffractometers (generator tension = 9 kV, current = 200 mA; anode Cu). The Atterberg limits of soil samples were determined following the USA Standard Test Methods-ASTM [36]. The vane shear strength of soil samples was examined on the laboratory vane apparatus (Wykeham farrance, 27-WF1730) following the British Standard BS-1377 [37] at the rotation rate of 6°/min.

## 3 Result and discussion

### 3.1 Removal efficiency of BPA

The application of PSF treatment is basically premised of cleanup of contaminants in soil. The removal of BPA by PSF treatment with various activator/enhancement has been studied extensively [38, 39]. Owning to the nonselective oxidation capacity of sulfate radical, part of Soil Organic Matter (SOM) could be removed as the persulfate treatment [40], subsequently may lead to the weakening of soil strength [41]. Therefore, it is significant to investigate removal efficiency of BPA and the changes of organic content after PSF treatment.

**Fig 1:**
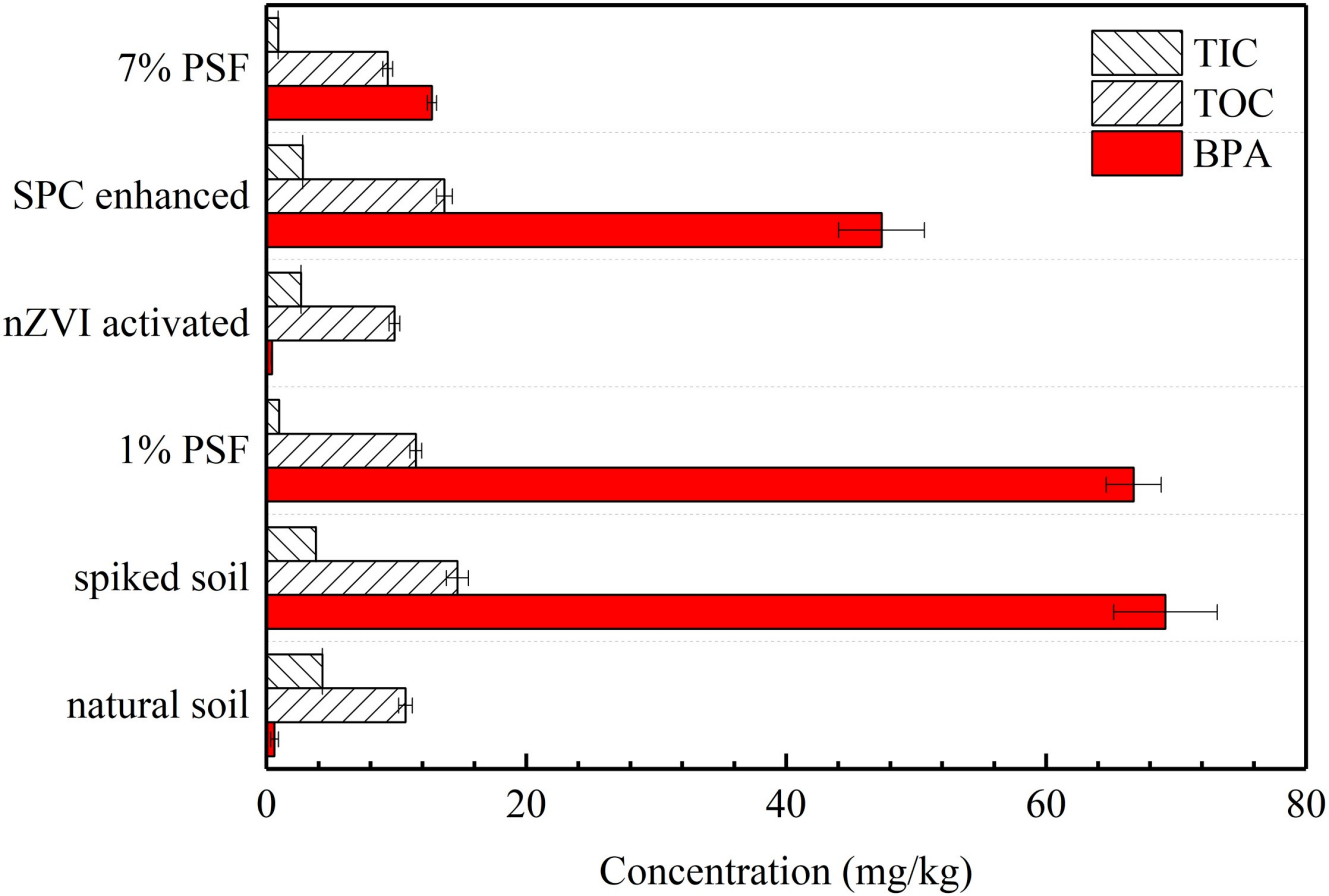
Removal efficiency of bisphenol A (BPA) and changes of Total Carbon (TC) by PSF treatment under different condition (TOC-Total Organic Compounds, TIC-Total Inorganic Compounds).

Fig 1 shows the removal efficiency of BPA and its changes of carbon content (TIC and TOC) by PSF treatment under different conditions. The removal efficiencies of BPA by different PSF treatment were followed by the order: nZVI activated >7% PSF>SPC enhanced >1% PSF. Interestingly, the PSF treatment at the dose of 1% led to a limited degradation of BPA, while higher dose of PSF (7%) resulted in a significant removal efficiency of BPA (i.e. about 80% removal efficiency). The mechanism for removal of BPA by pristine PSF treatment can be attributed primarily to the mineralization reaction by a range of reactive oxygen species by PSF that activated by some naturally occurring minerals (e. g. cobaltite, ilmenite, pyrite, and siderite) [42], some metal ions (e.g. Cu^2+^ or Fe^2+^ ions) [43], or the artificial itself [44–46] in the soils. The removal efficiency of BPA by PSF activated by nZVI presented the highest one, which is up to 99.9%. The higher removal efficiency using nZVI as activator than that of un-activated PSF treatment has been confirmed by many studies [47, 48]. As a contrast, the soil treated by PSF with SPC activated exhibited an unsatisfied removal efficiency.

On the other hand, the Total Carbon (TC) content in the soil were also impacted during the oxidative reaction involved in the PSF treatment. Due to artificial addition of BPA, the TC of the soil increased significantly. However, both Total Organic Compounds (TOC) content and Total Inorganic Compounds (TIC) content decreases at different levels after PSF treatment. The decrease of TOC can be attributed to the mineralization of these some organics with a relatively simple chemical structure such as BPA that been transformed into CO_2_ or CO_3_^2-^.

For the 1% PSF treated soil and nZVI activated soil, their resultant TC content is similar, while the BPA removal efficiency of nZVI treated soil is much higher than that of 1% PSF treated soil. A speculation is proposed for this difference that some organics with a relatively simple chemical structure such as BPA were easily degraded under the PSF/nZVI system within a short time, resulting in a decrease of the TOC content and an increase in TIC compared with that of 1% PSF treated soil. Meanwhile, the nZVI particles were passivated directly in the exposure of PSF and leading to the equal TC. This speculation is proposed based on the study reported by [49] that the nZVI particles can be passivated rapidly in the persulfate solution resulting the reaction rate to decrease to a level of a non-activated persulfate treatment case. Accordingly, the slightly increase of TIC in the PSF treatment with SPC enhanced one can be ascribed to the addition of percarbonate and its subsequent transition.

### 3.2 Particle size distribution

The particle size is one of essential physical parameters of soils that impact the soil strength [50]. In this study, the soil particle size distribution (PSD) of soil samples was calculated by laser diffraction technique, that is a well-established validity method [51].

**Fig 2:**
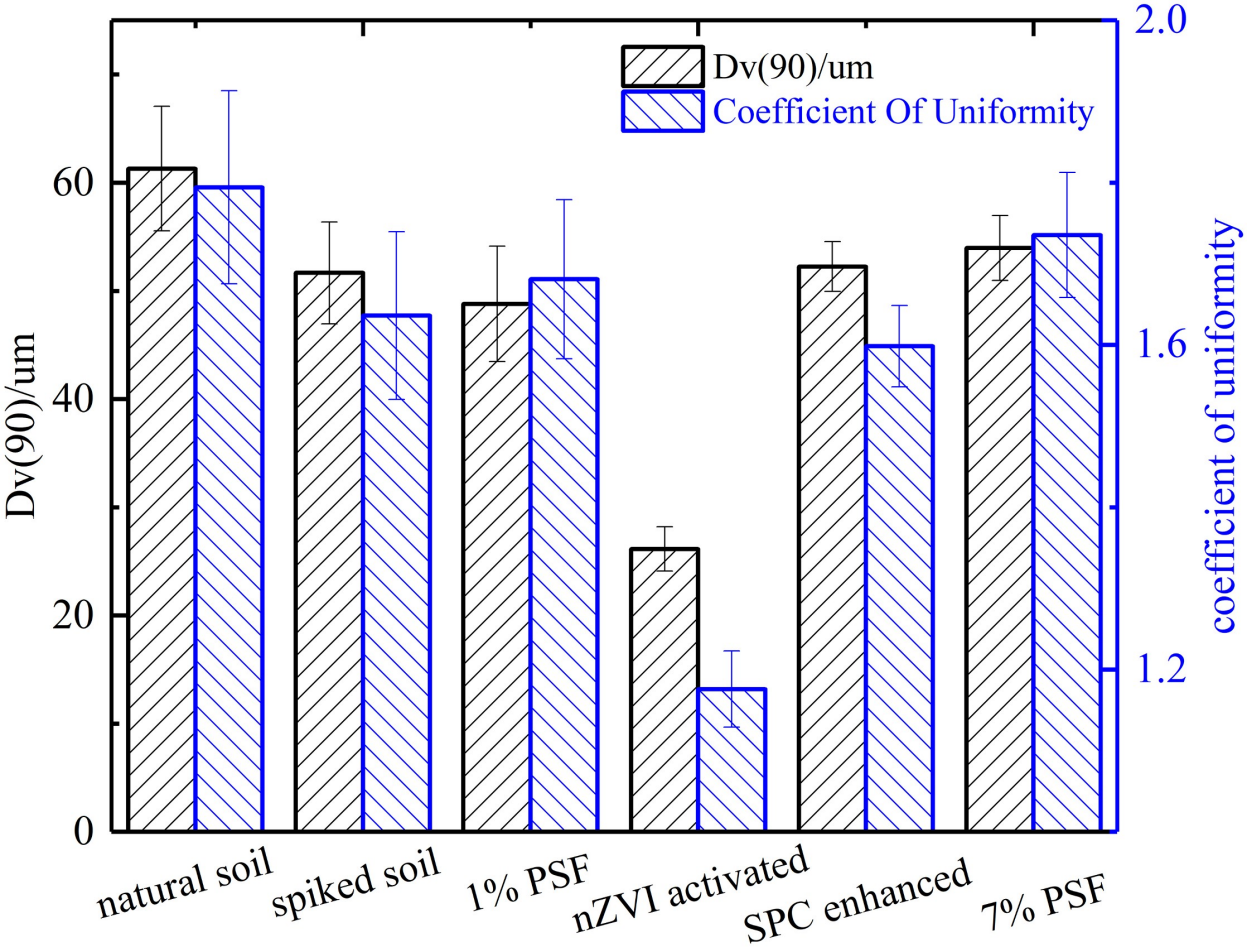
The changes of soil average particle size (Dv(90)) and the soil coefficient of uniformity.

**Fig 3:**
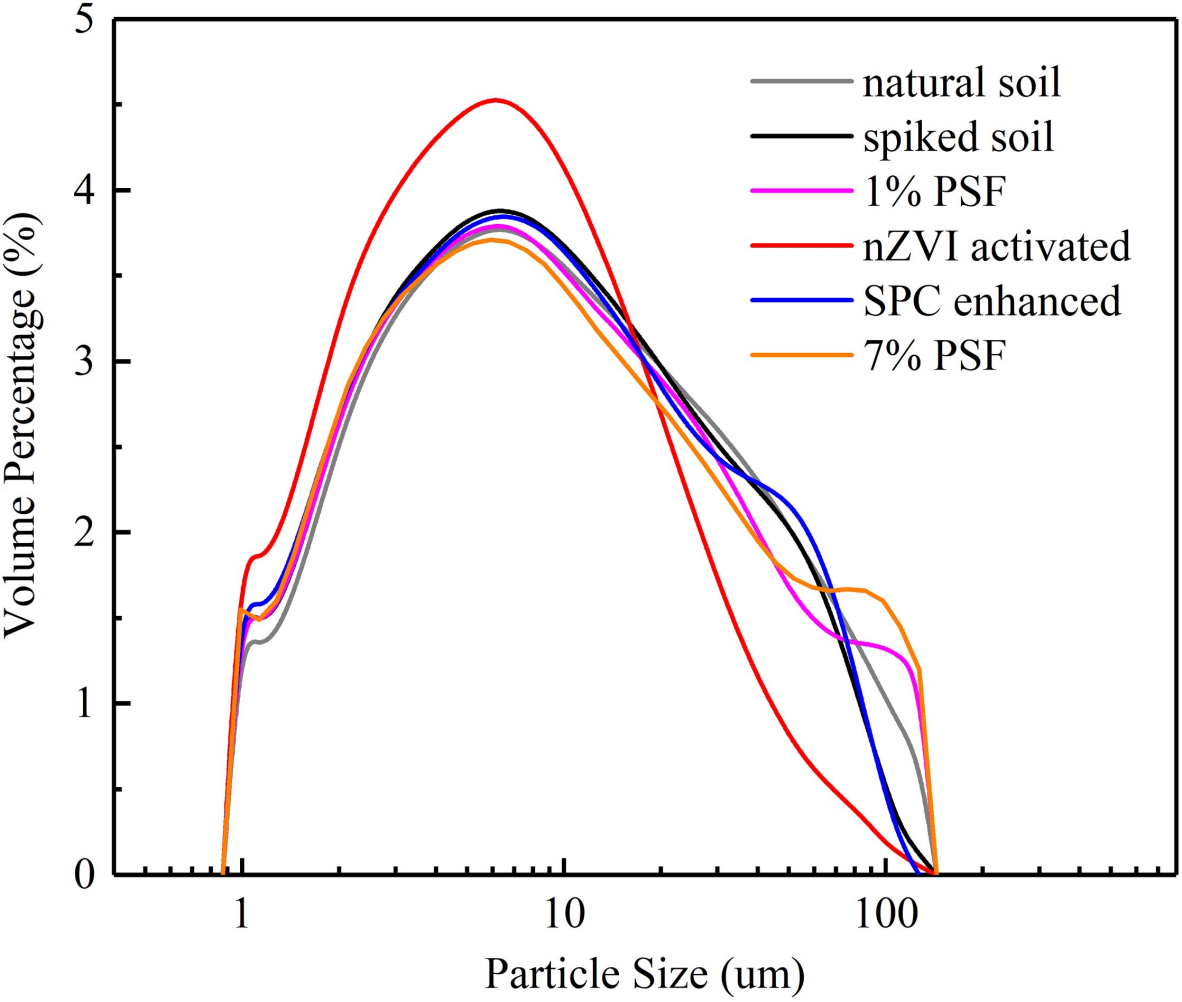
Particle size distribution of soil samples.

As is shown in Figs 2 and 3, the Dv(90) of the soil was 61.3 um. It is interesting that there were no remarkable changes in the soil average particle size less than around 10 um (i.e. the peak volume percentage) after contamination and treatment except the nZVI activated soil. As for the range from 10 um to 200 um, the PSD curve of BPA contaminated soil presents a similar tendency with that of SPC samples, which could conjecture that SPC have little effect on the soil particle size.

It can be seen that the percentage of the large particle size (>50 um) increase with the dosage of persulfate. The PSF treatment with nZVI activation resulted in a substantial decrease in the average size of soil particle. On the other hand, the uniformity and average particle size of soil samples present inconspicuous change except the nZVI activated soil sample. Both the soil uniformity and average particle size of nZVI activated soil decreased significantly compared with other soil samples. It is speculated that the introduced nZVI be oxidized rapidly in the soil slurry and swelled itself particle size from nano-scale to quasi micron-scale, which led to this prominent change.

**Fig 4:**
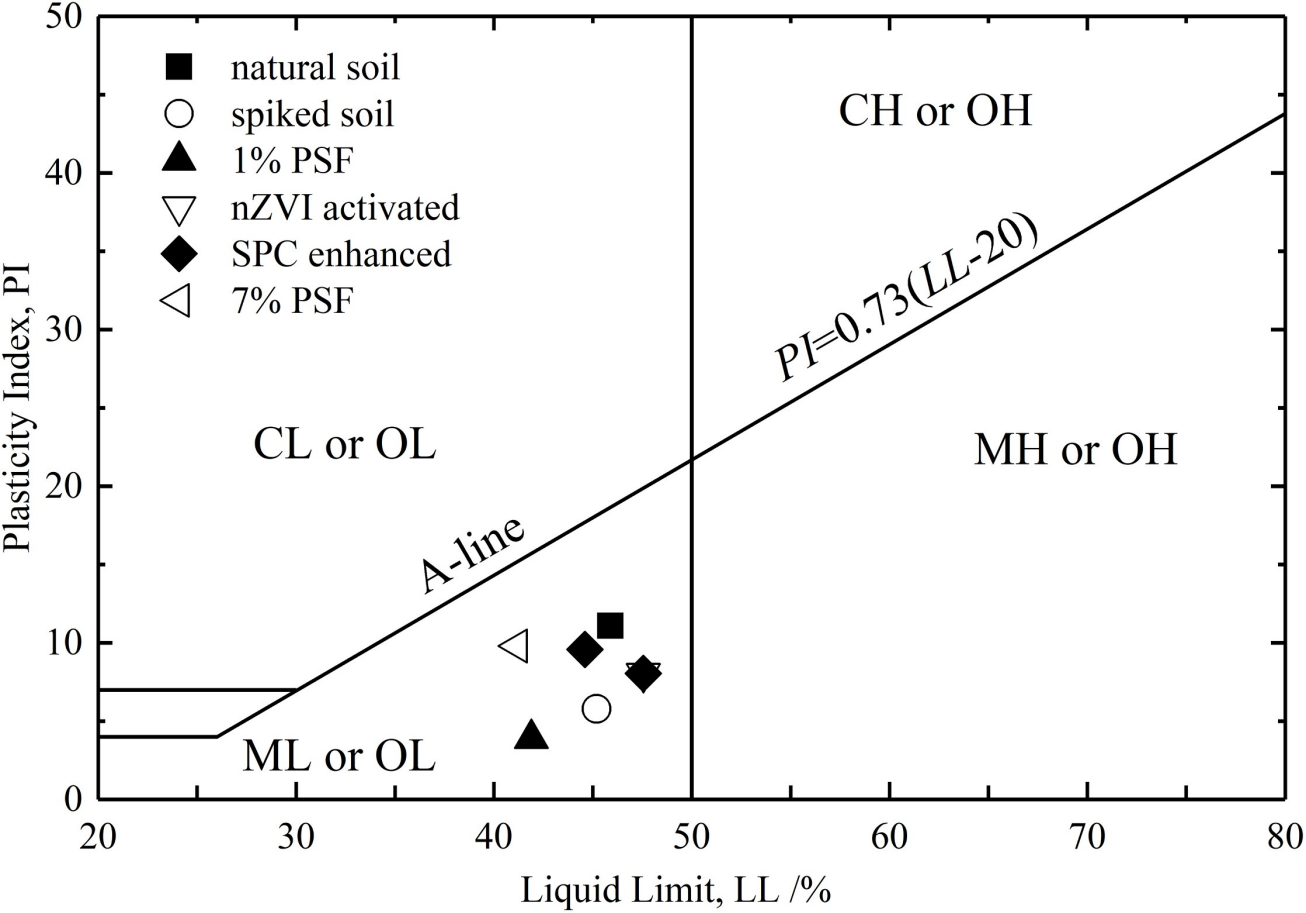
Classification of soil samples in the unified soil classification system.

The USA standard method ASTM D2487-17 was employed to describe the texture and grain size of the soil after different treatment. As is shown in Fig 4, all the soil samples are identified as silt (ML) or organic silt/clay (OL). Although the introduction of nano-sized iron particles changed the PSD significantly, it didn’t change the soil’s properties as ML or OL.

### 3.3 Soil buffering capacity

Buffering capacity is the intrinsic property of soils that quantifies the ability of a soil to resist changes in pH value. The change of pH value impacts the geotechnical properties of soil via influencing the chemical characteristics of soil, including mineralogical composition, ion exchange, ions adsorption competition, and surface precipitation [52–55]. Hence, it is significant to reveal the soil buffer capacity of the treated soil.

**Fig 5:**
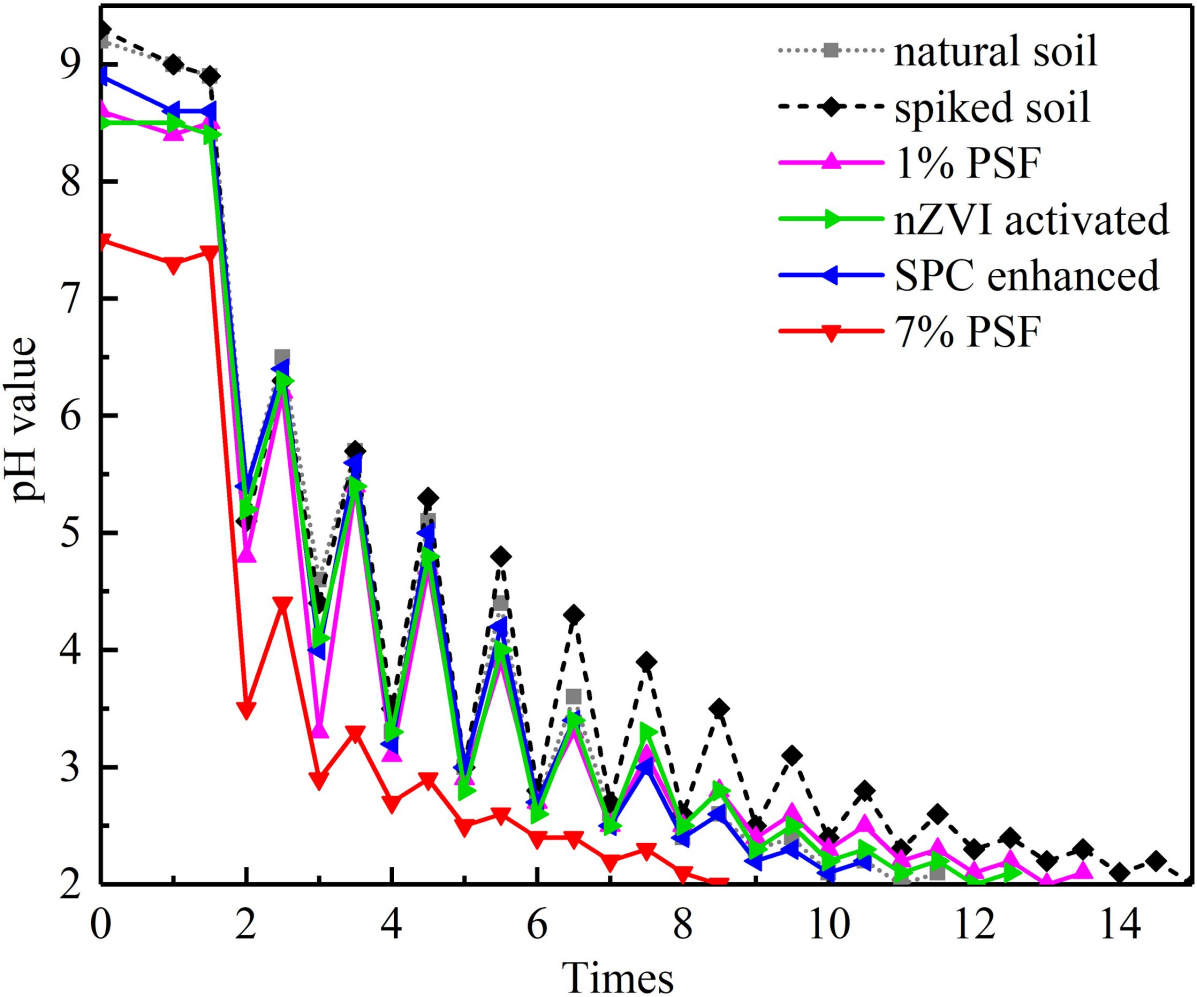
Acid buffering capacity of soil samples (100 uL 5 mol/L HCl at an interval time of 5 mins).

Fig 5 shown the acid buffering capacity of soil samples obtained by titration method. Results indicate that the natural soil used in this study is alkalescent. The introduction of BPA did not significantly change the soil pH value but improved the acid buffering capacity of the soil. For the soil samples treated by PSF with or without activation/enhancement, decreasing soil pH value and acid buffering capacity at different degree were confirmed. As a comparison, the PSF treatment at a higher dose (7%) resulted in a substantial drop of soil pH value and poor buffering capacity. This can be a disadvantage regarding its acid rain erosion durability, which may lead to the potential attenuation of mechanical properties in the long-term [56]. On the other hand, the changes of pH value buffering capacity are generally in agreement with their changes of TC in soils due to different treatment. The mineralization of natural organic compounds in soil may responsible partially for the declining soil pH value and poor buffering capacity.

### 3.4 Cation ions in pore fluid

**Fig 6:**
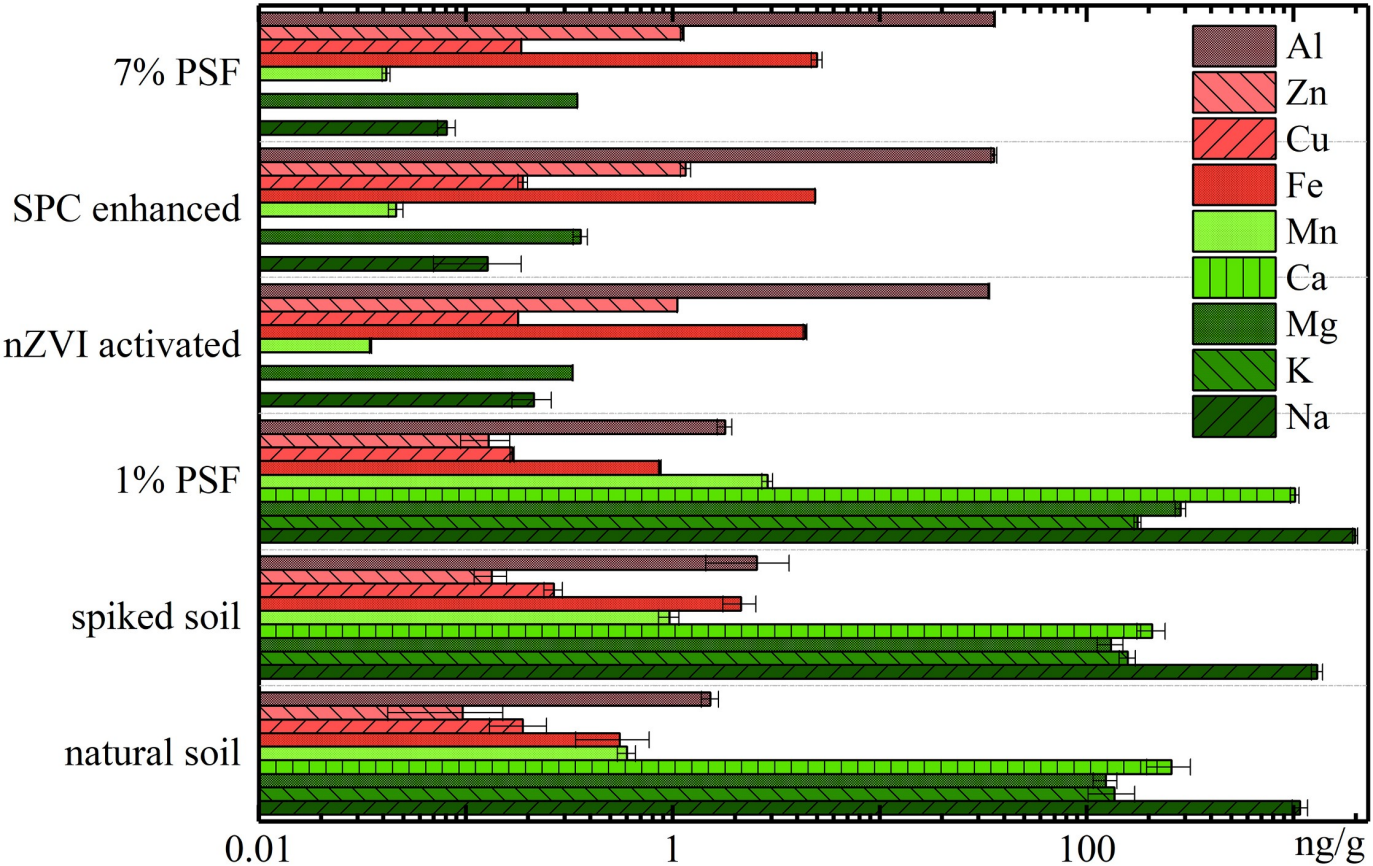
Concentration level of typical cation ions in soil pore fluid with error bars representing relative standard deviations (n = 2).

Many publications have indicated that cation ions in the pore fluid have different influences on the geotechnical properties of soil [57]. In this study, the typical cation ions in soil pore fluid including Al^3+^, Zn^2+^, Cu^2+^, Fe^3+^, Mn^2+^, Ca^2+^, Mg^2+^, K^+^, and Na^+^ were evaluated, as shown in Fig 6.

The results indicated that the K^+^, Na^+^, Mg^2+^, and Ca^2+^ are the dominant cation ions in the pore fluid of natural soil. The BPA contamination and PSF treatment at a dose of 1% did not lead to the significant concentration change of cation ions in pore fluid. Interestingly, remarkable changes of the concentration level of cation ions in nZVI activated, SPC enhanced, and 7% PSF treated soil samples were recorded. Two distinguished trends were observed. The concentration level of Mn^2+^, Ca^2+^, Mg^2+^, K^+^, and Na^+^ cations decreased sharply in these three soil samples. As a comparison, distinct increased concentration level of Al^3+^, Zn^2+^, and Fe^3+^ was noted. Additionally, no obvious changes were found on the concentration of Cu^2+^ in all soil samples.

The changes of cation ions concentration can be ascribed to the complicated and linked physicochemical reaction as a result of the introduction of PSF, nZVI, and SPC, in which desorption, adsorption, replacement, (co)precipitation, and oxidation are involved. For instance, the introduction of PSF and its produced massive sulfate ions can easily combine with the exchangeable Ca^2+^ ions in pore fluid for the formation of calcium sulfate (CaSO_4_) precipitation. Meanwhile, part of desorption of Ca^2+^ from unstable compounds on the soil particles may occur considering the excessive sulfate ions. The Na^+^, K^+^ and Mg^2+^ions will subsequently fill up the unoccupied adsorption sites, and lead to their significant decreases in the pore fluid. While the increased concentration of heavy metals (e. g. Al^3+^, Zn^2+^, and Cu^2+^) was attributed to the oxidation of unstable compounds by PSF or SPC [58]. The above results indicate that PSF involved AOPs can result in the evident changes of pore water chemistry. Because no significant variation was found on the concentration level of anions (sulfate, carbonate, nitrate, and chloride ions), we highlighted the changes of cations rather than anions in the pore fluid.

### 3.5 X-ray diffraction

**Fig 7:**
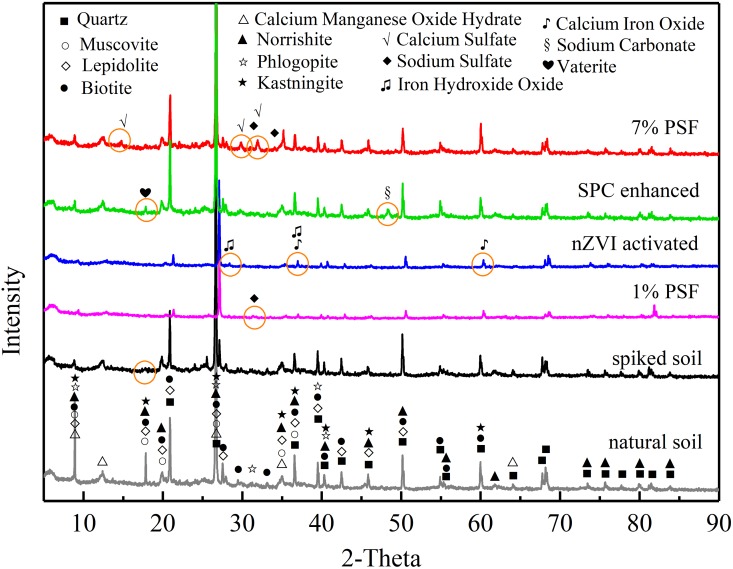
Results of X-ray diffraction (XRD) analyses.

X-ray diffraction (XRD) was employed to determine the changes of mineral component in the soil samples after different treatment. As is shown in Fig 7, analysis of XRD patterns indicated the complex mineral components in the natural soil. Quartz (SiO_2_, PDF#79–1906), Calcium Manganese Oxide Hydrate (Ca_2_Mn_14_O_27_·xH_2_O, PDF#50–0015), Muscovite (K, Na)(Al, Mg, Fe)_2_(Si_3.1_Al_0.9_)O_10_(OH)_2_, PDF#07–0042), and Lepidolite KMg_3_AlSi_3_O_10_OHF (PDF#73–1660) are the dominant minerals of natural soil. These mineral components are similar to the studied soil reported by Yan et al. (2010) and is consist with its pore water chemistry (Fig 6). Peaks figuring at the low angle ranges can be ascribed to the presence of organic matters in the soil [59]. The missing of characteristic peak at the 2-Theta of approximately 18 in the spiked soil sample suggests that the adsorbed BPA was potential corrosive to the soil that resulted in the alteration of the crystal structure of some minerals in soil [60–62].

As a comparison, the introduction of PSF, nZVI, and SPC led to the significant changes of characteristic peak (as circled in the Fig 6). This occurrence indicates that these oxidants (PSF and SPC) and reductant (nZVI) were of capabilities to change the mineral components in soil. After the PSF treatment, the presence of several fresh characteristic peaks at 2θ of 14.6, 29.7, 32.0, and 31.9, 34.0 can be ascribed to the generation of Calcium Sulfate (CaSO_4_, PDF#83–0437,) and Sodium Sulfate (Na_2_SO_4_, PDF#79–1553), respectively. The oxidation of nZVI led to the generation of Calcium Iron Oxide (CaFe_2_O_4_, PDF#71–2108, at 2θ of 37.0 and 60.5), Iron Hydroxide Oxide (FeOOH, PDF#70–0714 at 2θ of 28.6, 36.9, and 60.6), while the introduction of percarnonate resulted in the generation of Sodium Carbonate (Na_2_CO_3_, PDF#86–0295, at 2θ of 48.4), Vaterite (CaCO_3_, PDF#72–1616, at 2θ of 18.0). The generation of these additive-derived minerals may alter the geotechnical properties of soil in different ways.

### 3.6 Vane shear strength

**Fig 8:**
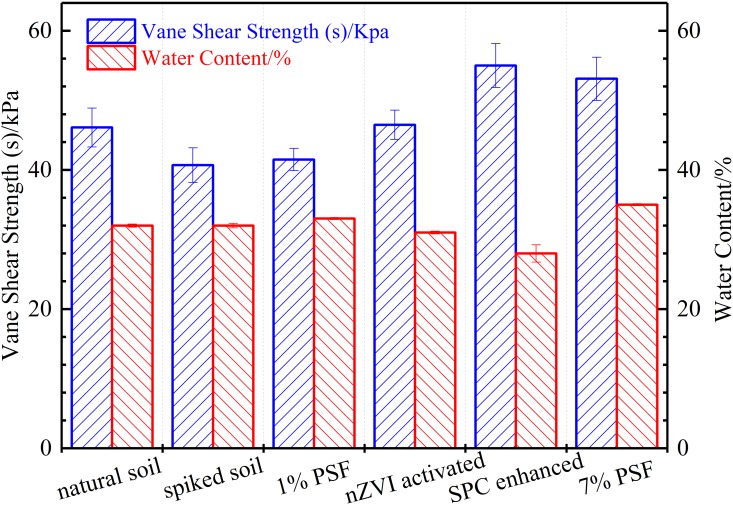
Vane shear strength and water content of soil samples after different PSF treatment with error bars representing relative standard deviations (n = 5 for vane shear strength and 2 for water content).

The vane shear test (VST) is an express and effective method that has been used widely to evaluate the undrained shear strength of cohesive soil [63, 64]. In this study, the laboratory vane shear test was employed to assess the changes of undrained shear strength of soil samples after different treatment of PSF (as shown in Fig 8 and Table 1). The results indicated that the artificial contamination using BPA led to a slight decrease in undrained shear strength. After the PSF treatment, the undrained shear strength of BPA-contaminated soil simultaneously increased at various degree. A wide thinking is proposed that both the non-activated PSF and the additives (nZVI and SPC) contributed to the soil improvement.

**Table 1:** Engineering properties of soil samples after different treatment.

Soil propertied	Natural soil	BPA spiked soil (spiked soil)	Spiked soil treated by 1% persulfate (1% PSF)	Spiked soil treated by 1% persulfate & 0.5% nZVI (nZVI activated)	Spiked soil treated by 1% persulfate & 0.5% SPC (SPC enhanced)	Spiked soil treated by 7% persulfate (7% PSF)
Liquid Limit (LL)	45.9	45.2	51.19	47.56	44.6	41.9
Plastic Limit (PL)	34.8	39.4	41.4	39.5	35	38
Plastic Index (PI)	11.1	5.8	9.79	8.06	9.6	3.9
Water content (%) ^a^	32.8	32.2	34.6	31.38	27.9	33.6
Weight of Soil samples (g) ^b^	183.5	182.0	174.6	173.6	182.3	181.7
Density (g/cm^3^)	1.93	1.91	1.83	1.82	1.91	1.91
Void ratio	0.86	0.87	0.98	0.95	0.8	0.89

Note:

^a^ refers to the water content of soil sample after pre-consolidation;

^b^ soil samples were trimmed at a height of 84 mm and diameter of 38 mm.

For the non-activated PSF treatment, it is reported that massive sulphate ions, the ultimate by-products after the mineralization of organic compounds by sulphate radical, were generated and retained in the soil [15]. The alkalescent environment as well as the presence of some cations (e.g. Ca^2+^) in the soil enabled the formation of sulfate deposition of calcium sulfate (CaSO_4_) precipitations on the surface of soil particles [65, 66]. This situation is consisting with the result of XRD as well as the changes of pore water chemistry. Several literatures also reported a similar positive effect of sulfate ions on mechanical properties of bonding material [67–69]. The PSF treatment used to remedy soil and its retained sulfate ions failed to show sulphate attack (namely deterioration of soil strength). It can attribute presumably to the low dose of concentration of sulphate ions used and the relatively large-sized silica particles in this study [70, 71]. On the other hand, the vane shear testing results suggested that the application of PSF treatment in relative low dose can achieve the removal of BPA and soil improvement simultaneously.

In addition to the above persulfate induced sulfate precipitation, nZVI and SPC also involved synergistically in the simultaneous removal of BPA and soil improvement. Nasehi et al. (2016) demonstrated that the nZVI treatment resulted in the cleanup of oil-contaminated clay as well as increase in water retention capacity, maximum dry density, and shear strength [72]. Similar results were also confirmed by our previous study [26]. A primary mechanism for the soil improvement using nZVI was that the pozzolanic-like reaction products contributed to heterogeneous flocculation by binding adjacent soil particles together and strengthen the soil. As for the SPC in the soil improvement, it is similar to the soil improvement using MICP that artificially adding chemical additives (Sodium Percarbonate) to increase the carbonates concentration in soil so that to form precipitation (Vaterite). This precipitation is capable of binding the soil particles side by side and leading to enhanced soil strength and stiffness. As a matter of fact, this process is similar to the Microbially Induced Calcite Precipitation (MICP) for soil improvement. The method of MICP employs biological reaction rather than chemical to produce carbonate in a calcium-rich environment, subsequently lead to the precipitations of CaCO_3_ and ground improvement [73, 74]. This also indicates that the pore water chemistry has considerable influence on the soil strength regarding the PSF involved remediation technology.

The water content of a soil is considerable in regulating the shear strength of soils, which generally present a negative relationship with its the shear strength [75]. In this study, the water content of soil treated by non-activated PSF slightly increased with the increase of PSF dose. The water content of soil samples treated by PSF activated by nZVI and SPC dropped slightly. There are two primary reasons for the changed water content: 1) different chemical compounds (e. g. iron hydroxides, calcium persulfate, calcium carbonate) were generated on the surface of soil particles or in the pore water; 2) the drainage condition was changed by the variational soil texture and components. Changes of water content and soil particle size did not present a specific pattern with varying Plastic limits (PL) and Liquid limits (LL) as well as shear strengths.

## 4 Conclusion

Sulphate ions are the primarily by-products in the application of PSF involved ISCO and are aggressive environmental deteriorations that can impact the soil strength by changing the microenvironment of soil. Their intruding in the subsurface may pose geoenvironmental risk to the redevelopment of the contaminated sites or the reuse of contaminated soil. In this study, we presented an investigation on the geoenvironmental characteristics of bisphenol A contaminated soil after persulfate treatment with nZVI and SPC activated/enhanced. Several conclusions were drawn based on the batch experiments:

1. The introduced PSF can be activated by some naturally occurring minerals, some metal ions or the artificial contaminant (BPA) itself and subsequently lead to the mineralization of BPA.
2. PSF involved AOPs can result in the evident changes of pore water chemistry, both Mn^2+^, Ca^2+^, Mg^2+^, K^+^, and Na^+^ cations decreased sharply in the soil pore fluid. The Ca^2+^ in pore fluid has considerable influence on the soil improvement in the PSF involved remediation technology.
3. The treatment of pure PSF, nZVI-activated and SPC-enhanced PSF can achieve removal of BPA and soil improvement simultaneously in the short-term. The primary mechanism of soil improvement can be ascribed to the heterogeneous deposition of by-products (calcium sulfate and/or calcium carbonate) on the surface of soil particles, subsequently bonding adjacent soil particles together and strengthen the soil. However, the soil buffering capacity decreased with the increase of PSF dose, which may impact the long-term service of PSF treatment in soil enhancement.

Considering the extensive application of PSF treatment in ISCO as well as the massive soil to be environmentally treated, the results of this study could make an important contribution to the remediation and redevelopment of brownfields and the reuse of contaminated soil in various engineering applications to achieve a sustainable and low-carbon society. However, the long-term effect of remediation agents at both lower and higher concentration on the geoenvironmental properties of the contaminated soil and groundwater warrants more research. Moreover, the soil strength and the sulfate ion in the pore water should be monitored to ensure the security of constructions in a long-term process.

## Supporting information

S1 File Data underlying this study. (ZIP)
